# Assessment of the Acute and Subchronic Toxicity and Mutagenicity of *Sideritis scardica* Griseb. Extracts

**DOI:** 10.3390/toxins10070258

**Published:** 2018-06-24

**Authors:** Björn Feistel, Tankred Wegener, Piotr Rzymski, Ivo Pischel

**Affiliations:** 1 Finzelberg GmbH & Co. KG, Andernach 56626, Germany; bjoern.feistel@finzelberg.de; 2 Consulting HMP, Weinheim 69469, Germany; t.wegener@consulting-hmp.de; 3 Department of Environmental Medicine, Poznan University of Medical Sciences, Poznan 60-806, Poland; 4 Dr. Ivo Pischel Consulting, Rossbach 53547, Germany; ivopischel@aol.com

**Keywords:** *Sideritis scardica*, toxicity, mutagenicity, extract

## Abstract

*Sideritis scardica* Griseb. has a long history of collection from the wild as a traditional remedy for respiratory and gastrointestinal complaints. It has also been investigated for its promising pharmacological activities in the central nervous system. However, its toxicological data is entirely missing. This study investigated the acute and repeated-dose oral toxicity of a *S. scardica* 20% (*v*/*v*) ethanol extract in Sprague Dawley rats, and mutagenicity using the Ames test. No gross pathological abnormalities and no toxicity signs or mortality were detected in animals treated with the dose of 2000 mg/kg bw during 14 days of observation. The tested extract was assigned to category 5 of the GHS. To evaluate a repeated-dose toxicity, an extract has been tested over a 28-day period followed by a 14-day recovery period. No mortality and no changes in body/organ weight or food consumption have been observed. The no-observed-adverse-effect-level of the extract was determined at 1000 mg/kg bw. The results of Ames tests conducted on extracts of different polarity (water; 20% (*v*/*v*) ethanol; 50% (*v*/*v*) ethanol; n-heptane), were unequivocally negative. The study reveals no toxicity of *S. scardica* and no concerns for its mutagenic effects, supports its positive safety profile, and confirms the acknowledged traditional medicinal use in human.

## 1. Introduction

The *Sideritis scardica* Griseb. (Lamiaceae family) is a subalpine/alpine plant species endemic to the central part of the Balkan Peninsula, specifically the northeastern regions of Greece, central and western regions of Macedonia, the southern regions of Bulgaria, and southwest regions of Albania [1,2]. Commonly known as ironwort, it has a long history of collection from the wild and exploitation for its aromatic properties in local cuisines as a flavour or a tea substitute for recreational use (known as mountain tea or shepherd´s tea), otherwise also for the treatment of certain complaints of the respiratory (e.g., asthma, cough, bronchitis, lung emphysema) and gastrointestinal tracts, and for immunomodulating and diuretic action that would yet require clinical evidence. Moreover, its ethanolic extracts are also traditionally used as an oral cavity antiseptic [2]. Beyond such use, pharmacological activity of *S. scardica* in the central nervous is currently thoroughly studied. It has already been evidenced in vitro that alcoholic *S. scardica* extracts can affect uptake of monoamine neurotransmitters [3]. Recent in vivo studies highlighted that oral intake of *S. scardica* enhances cognition in aged, nontransgenic as well as in APP-transgenic mice, can reduce amyloid-β load, increase phagocytosis of microglia and expression of secretase ADAM10, and fully rescue neuronal loss to normal levels [4,5]. These findings advocate a potential use of *S. scardica* extracts in the treatment or prevention of Alzheimer’s disease and highlight a need for further studies in this field. The investigations to ensure the safety of *S. scardica* use are also urgently needed.

Hitherto, there are no registered or authorized medicinal products in the European Community containing *S. scardica* as an active ingredient [6,7]. The plant extract has been reported to contain monoterpenes, sesquiterpenes, diterpenes, triterpenes, sterols, flavones, coumarins, and phenylpropanoids [6,7,8,9]. Recently, the Committee on Herbal Medicinal Products of the European Medicines Agency (EMA) acknowledged the medicinal use of a tea made from *S. scardica* as a traditional herbal medicinal product used for the relief of coughs associated with cold and for the relief of mild gastrointestinal discomfort [4,5]. The *S. scardica* products are also available as food supplements or herbs, but as such, their registration requires neither preclinical or clinical studies [10,11,12].

Herbal drugs have been used for medicinal purposes since centuries ago, which is often well-documented in literature or from textbooks, and in some cases even in the first herbal books (Kreuter- or Kräuterbücher) of the 16th century or earlier. These data may be used during authorization or registration procedures as well-established use (WEU) or traditionally used (THMP) herbal medicinal products in the European Community, and might provide valuable information particularly in respect to therapeutic use, tolerability, and toxicity [13,14,15,16]. Attention should be paid to biological effects that are difficult to detect clinically. These include safety aspects related to reproductive toxicity, genotoxicity, and carcinogenicity, whereas especially the lack of genotoxicity data may present a concern [17].

There is no data on the safety endpoints of *S. scardica*. These endpoints are difficult to study in humans, although they are required to build up a safety profile. This is particularly relevant for data on the acute and repeated-dose toxicity and mutagenicity. The only available data is from the in vitro studies involving B16 cells and HL-60 cells, and indicates that highly lipophilic diethyl ether extract of *S. scardica* can exert moderate cytotoxic effects [18]. Considering that wild specimens of *S. scardica* are collected for consumption and that there is a growing evidence of the potential therapeutic use of its extracts in some neurodegenerative disorders, it is imperative to evaluate whether constituents of this plant do not exhibit health threats. Therefore, the purpose of the present study was to evaluate the preclinical safety profile of *S. scardica* extracts with respect to acute and repeated-dose oral toxicity and genotoxicity. The research was conducted according to the guidelines of the Organisation for Economic Cooperation and Development (OECD) [19,20,21].

## 2. Results

### 2.1. Phytochemical Analyses

The total phenolic content determined in the 20% EtOH (*v*/*v*) *S. scardica* extract amounted to 6.25%. The major phenolic classes were represented by flavonoids, followed by caffeoyl quinic acids and acteoside. The detailed information on their content in the studied extracts is given in Table 1.

**Figure 1:**
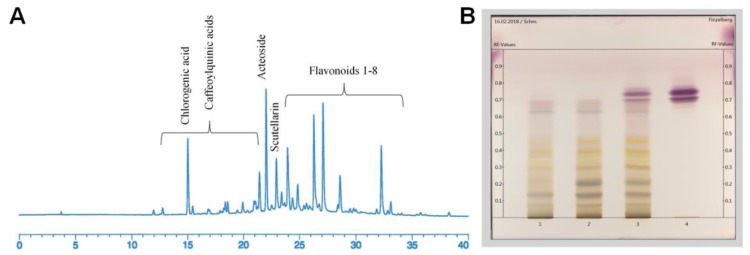
The results of phytochemical analyses of *S. scardica* extracts. (**A**) The HPLC fingerprint chromatogram of the 20% EtOH extract; (**B**) Thin-layer chromatogram (TLC) fingerprint for polar constituents according to the Finzelberg method. Lanes from the left represent the following extracts: 1: aqueous; 2: 20% EtOH; 3: 50% EtOH; 4: heptane.

The high-performance liquid chromatography (HPLC) analysis depicted several groups of plant constituents in the *S. scardica* extract with 20% ethanol (*v*/*v*) (Figure 1A). The phytochemical characterisation focused on the peaks between 15–22 min, representing phenolic acids and derivatives thereof, including several caffeoylquinoic acids, such as chlorogenic acid. These were followed by acteoside (peak at about 22 min), also known as verbascoside, chemically a phenylethanoid glycoside, and between the retention time 23 to 33 min, a larger group of flavonoids and their glycosides (such as scutellarin, apigenin, luteolin, and others; flavonoid structure confirmed by their UV spectra) were observed.

Whereas HPLC analysis only showed fractions of extract constituents, the fingerprints of the thin-layer chromatograms (TLC) revealed the compositions of the whole extracts obtained with the extraction solvents water and ethanol with increasing concentration (Figure 1B). Thus, extracts no. I, II, and III yielded polar constituents of *S. scardica*, which deliver intensive bands along the TLC tracks in the range of 0.1 to 0.65 of retardation factor (Rf) values. The lanes obtained for the water extract and 20% EtOH extract revealed the greatest similarity. In the presence of nonpolar heptane solvent, the polar substances were not extracted, but the lipophilic compounds, including fatty and waxy constituents and chlorophyll, were observed (Rf 0.7–0.8).

### 2.2. Acute Oral Toxicity

All animals survived the study period and no signs of toxicity or mortality were observed. Gross pathological examination did not reveal any abnormalities, thus no histopathological investigations were performed. The percent body weight gain after seven days was found to be 8.17 and 7.76% in the animals of step 1 and 2, respectively, and 17.81 and 17.58% after 14 days, respectively.

### 2.3. Repeated-Dose Oral Toxicity

All rats survived the study period and there were no differences between body weight gain and food intake. The daily observations did not reveal any signs of toxicity, altered behavior, or functional symptoms attributable to the treatment. After the dosing period on day 29, hematological analysis revealed no abnormalities attributable to the treatment except for a significant decrease of white blood cell count in males treated with 500 mg/kg. The hematological analysis in the reversal group on day 43 revealed no abnormalities attributable to the treatment (Table 2).

The biochemical analysis on day 29 revealed no abnormalities attributable to the treatment, except for significant increases of calcium concentration (250 mg/kg, 500 mg/kg, and 1000 mg/kg; males) and significant decreases of calcium (250 mg/kg and 1000 mg/kg, females) and sodium (500 mg/kg and 1000 mg/kg, females) concentrations. The analysis in the reversal group on day 43 revealed no abnormalities attributable to the treatment except for a significant increase of calcium in males with the 1000 mg/kg dose (Table 3). All these changes of hematological and clinical biochemical parameters were marginal and considered to be within the normal biological and laboratory limits. Thus, the no-observable-adverse-effect level (NOAEL) of the investigated dry extract was determined to be 1000 mg/kg body weight in male and female animals in this 28-day repeated-dose oral toxicity study.

### 2.4. Mutagenicity Tests

**Figure 2:**
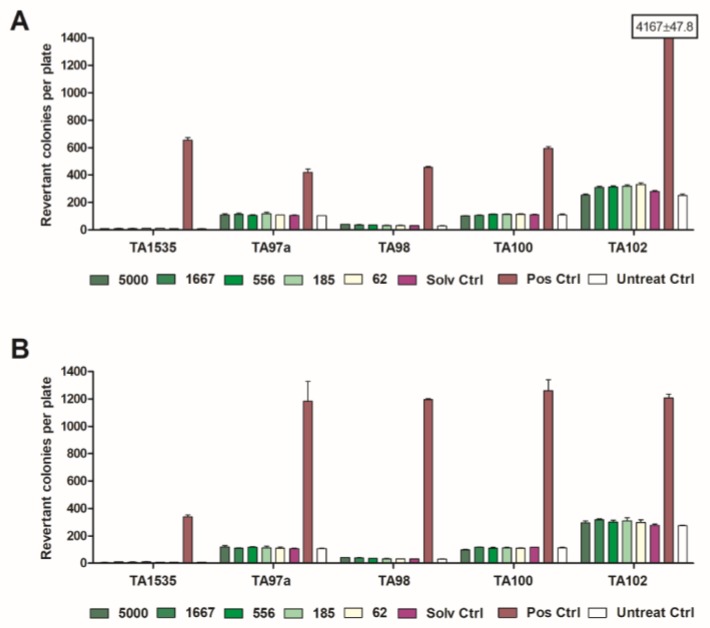
The results (mean ± SD) of the plate incorporation test without S9 metabolic activation (**A**) and with S9 metabolic activation (**B**) of extract I (20% EtOH), applied in concentrations of 5000, 1667, 556, 185, and 62 µg/plate (Solv Ctrl = solvent control, Pos Ctrl = positive control, Untreat Ctrl = untreated control). The effect was only significant (*p* < 0.05) in the case of the positive control.

The study revealed that the mean numbers of revertant colonies counted at different concentrations were comparable to those of the negative controls for both experiments, in the absence and presence of metabolic activation. In all tests, the positive controls (reference mutagens) induced the expected increase of revertant colonies, indicating the validity of all experiments (Figure 2). The results indicated that extract I (20% EtOH) tested at up to 5000 μg/plate concentrations did not induce any mutations in the *Salmonella* strains. The results of the test with this extract, expressed as the mean number of revertant colonies per plate with and without metabolic activation, are shown in Figure 2A,B, respectively. The exposure of all tested strains to extract II (water), III (50% EtOH), and IV (n-heptane) did not cause any biologically relevant increase in revertant colony numbers, both in the presence and absence of metabolic activation.

## 3. Discussion

Until now, there has been no comprehensive data on the toxicity of *S. scardica* except for one in vitro study, in which a significant dose-dependent decrease in proliferation was found in B16 (by 51.3%) and HL-60 (by 77.5%) cells treated with diethyl ether extract, and as speculated, phenolic compounds such as apigenin, luteolin, and their corresponding glycosides could be responsible for this effect [18]. These findings strongly advocated the need to further the toxicity evaluation of *S. scardica*, preferably using an in vivo experimental model. Considering that some plant species belonging to the Lamiaceae family (e.g., *Origanum majorana*) were already found to contain metabolites exerting genotoxic action [22], there remained a need to assess it in *S. scardica*, not only due to its traditional and frequent use as a food ingredient or natural medicine in the Balkan Peninsula region and the increasing popularity of food supplements based on dried *S. scardica*, but also because its extracts have been extensively studied for use in the treatment or prevention of neurodegenerative disorders, yielding highly promising effects [4,5]. The present study provides an information on acute and repeated-dose in vivo toxicity as well as mutagenicity, according to established regulatory guidelines. These results contribute to the evaluation of the safety of food supplements based on *S. scardica*, reassuring that health risks related to the traditional use of this plant in some European countries are unlikely, and also support the safety of the potential introduction of *S. scardica* extracts to clinical trials (which is plausible given the observed neuroprotective effects). Moreover, as shown, the investigated 20% EtOH extract contained polar constituents, particularly flavonoids, followed by caffeoylquinic acids and acteoside. The TLC analysis revealed similarities in composition between this extract and that obtained with water. The water extraction is in turn closely related to a tea preparation, which is a traditional form of the use of *S. scardica* [1,2].

To evaluate acute oral toxicity according to OECD Guideline 423 [21], the acute toxic class method (or limit test) was used. This method involves a stepwise procedure with the use of three animals of a single sex per step, and depending on the mortality and/or the moribund status of the animals, only a few further steps may be necessary to allow a judgment on the acute toxicity of the test substance. In our study with a dose of 2000 mg/kg bw, all animals survived the study period showing no signs of toxicity, mortality, or any gross pathological abnormalities. According to the Globally Harmonized System of Classification and Labeling of Chemicals (GHS), with five categories for acute toxicity, the tested extract is classified into category 5 (chemicals with oral and dermal LD_50_ values of 2000 to 5000 mg/kg bw), and thus may exert only a low toxicity.

According to OECD Guideline 407 [19], the test for repeated-dose oral toxicity may be carried out after initial information on toxicity has been obtained by acute testing. Such a study provides information on the possible health hazards likely to arise from repeated exposure over a relatively limited period of time, whereas the duration of exposure should normally be 28 days, as was done in the presented study. Here, all rats survived the study period without any differences between body weight gain, food intake, altered behavior, or functional symptoms attributable to the treatment between the groups. There were only some findings of minor relevance in respect to direct toxic effects (changes in white blood cell count and of concentrations of calcium and sodium, depending on sex and dose), which are hard to interpret as the effects are not directed. For example, the white blood cell count was decreased only in males treated with 500 mg/kg bw, but not in the group exposed to 1000 mg/kg bw, indicating that this effect cannot be considered to be caused by the administration of extract. Moreover, the lowest white blood cell count observed in the study (11.66 ± 3.11 × 10^3^/µL) fell within ranges observed for healthy Sprague-Dawley rats [23,24]. Calcium concentrations were, in turn, increased in treated males, but decreased in the female group, but in both cases did not reveal a dose-dependent relationship. However, a sodium concentration was decreased in females treated with 500 and 1000 mg/kg bw, and in both cases, the observed levels of this mineral fell within ranges observed for untreated Sprague-Dawley rats [24]. All changes of hematological and clinical biochemical parameters were marginal and considered to be within the normal biological and laboratory limits, and a NOAEL of extract I was determined to be at 1000 mg/kg bw in male and female animals in this study’s conditions.

In all Ames tests performed in the present study, no mutagenic effect measured by an increase of revertant colony numbers as compared to control counts was observed for any of the extracts tested up to concentrations of 5000 µg/plate. This is true for both independent experiments in each test with and without metabolic activation, including the plate incorporation as well as the preincubation method. The four different extracts I–IV were selected according to the principles of the “bracketing and matrixing” concept [25], and thus the entire spectrum of constituents of the *S. scardica* herb, including polar and nonpolar constituents, was involved in the mutagenicity testing. Therefore, it can be stated that in the described mutagenicity tests and under the experimental conditions reported, the herb of *S. scardica* and its constituents did not cause gene mutations by base pair changes or frameshifts in the genomes of the tester strains used.

## 4. Conclusions

Despite the long tradition of the use of *S. scardica* for food, as well as experimental studies on its medicinal use, there had been essentially no data available on systematic toxicity testing for an evaluation of its safety. In this study, a range of basic toxicity tests were conducted to explore the nonclinical safety of a dry hydroethanolic extract from the herb of *S. scardica*. In the test for acute oral toxicity, no gross pathological abnormalities and no signs of toxicity or mortality could be detected in rats up to a dose of 2000 mg/kg bw. Thus, the extract may be assigned to category 5 as the lowest class of toxicity according to the GHS. In a 28-day repeated-dose toxicity study in rats, the NOAEL was determined at 1000 mg/kg bw. Finally, in a set of Ames tests of various extracts of the herb, selected according to the “bracketing and matrixing” concept to include the full range of polar and nonpolar constituents, did not reveal concerns for mutagenicity. Overall, the present study results support a positive safety profile of *S. scardica* and indicate that its toxic action when used for various purposes is unlikely.

## 5. Materials and Methods

### 5.1. Selection of Extracts

All tested extracts were produced from the aerial parts of *Sideritis scardica* Griseb. (Herba; a voucher herbarium specimen of the taxonomically identified sample is deposited at the Department of Pharmacognosy and Natural Products Chemistry, Faculty of Pharmacy, University of Athens, Greece (Specimen-No. PAS101) and were provided by Finzelberg GmbH & Co. KG, Andernach, Germany, as homogenous drug powder. For the purpose of the present study, the following extracts were prepared by employing 10 kg of the botanical drug for the extraction with 10-fold quantities of extraction solvent (100 L):

- extract I (used for test of acute and repeated-dose toxicity and mutagenicity tests): dry extract made with 20% *v*/*v* ethanol as extraction solvent, drug/extract ratio (DER) native 5–9:1, 70% native extract, adjustment with 30% maltodextrin;
- extract II (used for mutagenicity test): dry extract made with water as extraction solvent, drug/extract ratio (DER) native 4–8:1, 70% native extract, adjustment with 30% maltodextrin;
- extract III (used for mutagenicity test): dry extract made 50% *v*/*v* ethanol as extraction solvent, drug/extract ratio (DER) native 6:1, 70% native extract, adjustment with 15% maltodextrin (MD) and 15% silica;
- extract IV (used for mutagenicity test): dry extract made with n-heptane as extraction solvent, drug/extract ratio (DER) native 83:1, 50% native extract, adjustment with 50% silica.

### 5.2. Phytochemical Analysis

The extracts were analyzed for total polyphenols with a Folin–Ciocalteu method following guidelines of the European Pharmacopoeia [18] and for specific polyphenolic compounds (flavonoids, acteoside, caffeoylquinic acids) with a high-performance liquid chromatography (HPLC) method. For this purpose, a LunaR C18/2 column (Phenomenex, Torrance, CA, USA; 250 mm length, 4.6 mm inner diameter, 5 μm particle size) was used at 40 °C in a Shimadzu LC10 HPLC system (Shimadzu Deutschland GmbH, Duisburg, Germany). Ten microlitres of 5 mg/mL sample was injected. The mobile phase consisted of water + 0.1% H_3_PO_4_ (solvent A) and acetonitrile + 0.1% H_3_PO_4_ (solvent B) with the following gradient: from 95% A/5% B (0 min) to 50% A/50% B in 41 min; 100% B from 45 to 50 min to 95% A/5% B until 52 min; 65 min in total. The compounds were detected by diode array detector at 330 nm and calculated through scutellarin and acteoside (Phytolab, Vestenbergsgreuth, Germany) as external standards.

Additionally, thin-layer chromatography (TLC) was conducted to highlight differences of the extract made by different polar extraction solvents. As the stationary phase, silica gel 60 F254 was used. The plate was cleaned and activated with ethyl acetate/methanol 50:50 (*v*/*v*) and dried at 105 °C for 30 min. Ten microlitres of preparations from 1 g of *S. scardica* extract and 10 mL EtOH 50% (10 min at 65 °C, filtered) were applied and separated within 15 cm using dichloroethane/acetic acid/methanol/water 50:25:15:10 (*v*/*v*/*v*/*v*) as the mobile phase. After drying, anisaldehyde solution R was sprayed on the plate, which was dried again for 3 min at 120 °C [26].

### 5.3. Acute Oral Toxicity Tests

The study for acute oral toxicity has been performed according to the OECD guideline for the Testing of Chemicals No. 423 [21] at the Indian Institute of Toxicology, Pune, India. Healthy young adult (8–12 weeks of age) female (nulliparous and nonpregnant) Sprague-Dawley rats with uniform body weight (within ±20% of the mean) provided by National Institute of Biosciences, Thane, India, were used and acclimatized to laboratory conditions in single polycarbonate cages for a minimum of 5 days. They were maintained at a controlled temperature of 19.5–21.8 °C, relative humidity of 48.4–55.8%, and 12-h light/dark cycle. Animals were fed a rodent feed supplied by Pranav Agro Industries Ltd., Sangli, India, and allowed osmosis water from a local source, both ad libitum. A single dose of 2000 mg/kg bw of the freshly prepared extract I in distilled water (adjusted strength of 200 mg/mL, dose calculation 10 mL/kg of fasting body weight) was administered by oral gavage to three animals, followed by 14 days of observation (step 1). As there were no signs of any toxicity or mortality, three further female animals were added to the study and treated with the same dose (step 2). The applied dosage was selected based on data from the literature and from the knowledge of the phytochemical composition of the test item [1,6,7].

In each step, the animals have been controlled for clinical signs and mortality immediately after application at 5, 10, 30, and after 60 min as well as after 2, 4, and 6 h on the day of dosing, and followed daily for 14 days until sacrifice. Individual body weights were recorded prior to test item administration and thereafter weekly until study termination. All animals surviving the study period were sacrificed by carbon dioxide asphyxiation technique on day 15, followed by necropsy. OECD/EU-GLP approval code: Registration No: I01 by WIV-ISP, Brussels, Belgium, Date of approval: 4 January 2010, and Certificate No.: GLP/C-022 by National (Indian) GLP Compliance Monitoring Authority, New Delhi, India, Date of approval: 16 April 2010.

### 5.4. Repeated-Dose Oral Toxicity Tests

The study for 28-day oral toxicity has been performed according to the OECD guideline for Testing of Chemicals No. 407 [19] at the Indian Institute of Toxicology, Pune, India. Male and female (nulliparous and nonpregnant) 5–6-weeks-old Sprague Dawley rats with uniform body weight (within ±20% of the mean) provided by National Institute of Biosciences, Thane, India, were used and acclimatized to laboratory conditions in polycarbonate cages for a minimum of 5 days. They were maintained at a controlled temperature of 22 ± 3 °C, relative humidity of 30–70%, and 12-h light/dark cycle, fed with a rodent feed supplied by Nutrivet Life Sciences, Pune, India, and allowed osmosis water from a local source, both ad libitum. A total of 60 animals (30 males and 30 females) were randomly allocated to six groups with 5 animals per sex and dose group, and after dosing, were housed with 5 animals per sex and per cage. Over 28 days, the animals were administered doses of 0, 250, 500, and 1000 mg/kg bw of extract I per day by oral gavage (adjusted strength of 200 mg/mL, dose calculation 10 mL/kg of fasting body weight). The dosages were selected based on the findings of the study on acute toxicity. The animals were observed twice daily throughout the study period of 28 days, and animals of additional dose groups of 0 and 1000 mg/kg doses for a further 14-day post-treatment period (reversal groups; included to study reversibility or delayed occurrence of symptoms). During the study, all animals were monitored for clinical, behavioral, and functional symptoms. The hematological, biochemical, and electrolytical parameters were evaluated for all animals scheduled to be sacrificed at the end of dosing on day 29 or day 43 in the case of the reversal group. The following hematological parameters were evaluated via Beckman Coulter Hematology Analyzer (Beckman Coulter, Brea, CA, USA): haemoglobin (Hb), erythrocyte count (RBC), haematocrit (HCT), mean corpuscular volume (MCV), mean corpuscular haemoglobin (MCH), mean corpuscular haemoglobin concentration (MCHC), platelet count (PLT), and total white blood cells (WBCs). Differential leukocyte counts were performed manually from microscopic specimens. Prothrombin time (Pt) was measured using citrate bulb (100 μL of 3.8% solution of sodium citrate per mL of blood). The following serum biochemistry parameters were studied using VeTEX Veterinary Chemistry Expert (Wipro Biomed, Bangalore, India): alanine aminotransferase (ALT), aspartate aminotransferase (AST), alkaline phosphatase (AP), glucose, total bilirubin, albumin, globulin, creatinine, total cholesterol, triglycerides, and urea. The electrolyte analyses (calcium, phosphorus, sodium, potassium, chloride) were performed with Acculyte P5 (Aspen Diagnostics, Delhi, India). For pathological (necropsy, organ weights, and gross pathology) investigations, all animals were sacrificed on day 29 or day 43 (reversal groups) using a carbon dioxide asphyxiation technique. OECD/EU-GLP approval code: Registration No: I01 by WIV-ISP, Brussels, Belgium, Date of approval: 4 January 2010, and Certificate No.: GLP/C-022 by National (Indian) GLP Compliance Monitoring Authority, New Delhi, India, Date of approval: 16 April 2010.

### 5.5. Mutagenicity Test (*Salmonella typhimurium* Reverse Mutation Assay)

Mutagenicity of all dry extracts (I–IV) had been evaluated with the Ames test (*Salmonella typhimurium* reverse mutation assay, the most widely used short-term and thoroughly validated test for the identification of carcinogens using mutagenicity in bacteria as an endpoint [27]. In the present study, the testing was conducted according to the relevant OECD guideline [20] (extracts I–IV) and other recommendations and guidelines [28,29] (extracts II–IV). The use of extracts I–IV was selected according to the principles of the “bracketing and matrixing” concept of Guideline EMEA/HMPC/67644/2009 [25] and represented the complete range of extraction solvents, including water as the most polar and heptane as the most nonpolar solvent. The tests with extract I were conducted at the Indian Institute of Toxicology, Pune, India; and the tests with extracts II–IV at Bioservice Scientific Laboratories GmbH, Planegg, Germany. Each test was conducted in two independent experiments with plating in triplicates at each concentration.

Extract I was tested with *S. typhimurium* strains TA97a, TA98, TA100, TA1535, and TA102 with and without metabolic activation by rat liver S9 mix. The extract was tested at doses of 61.72, 185.18, 555.55, 1666.67, and 5000 μg/plate in the plate incorporation method. Extracts II–IV were tested with *S. typhimurium* strains TA98, TA100, TA1535, TA1537, and TA102 with and without metabolic activation by rat liver S9 mix. All extracts were tested at doses of up to 5000 μg/plate in the plate incorporation and in the preincubation method.

For testing, the extracts were dissolved in dimethylsulfoxide (DMSO); the test on cytotoxicity was carried out with *S. typhimurium* strain TA100 with and without metabolic activation; and the metabolic activation was made with post-mitochondrial fraction (S9 fraction) from metabolically activated rats.

For the plate incorporation test, each of the extracts I–IV were blended with or without S9 mix into a soft agar and immediately poured onto a minimal glucose agar plate. Plates were inverted and incubated at 37 °C for 48 to 72 h. For the preincubation method, conducted with extracts II–IV, they were preincubated with the test strain for 20 min at 37 °C prior to mixing with the overlay agar. The remaining steps were the same as described for the plate incorporation method. The revertant colonies on the test and control plates were counted manually (test with extract I) or with a ProtoCOL colony counter (Meintrup DWS Laborgeräte GmbH, Herzlake, Germany; test with extracts II–IV).

For testing of extract I, positive controls (Pos Ctrl) were performed without metabolic activation using known mutagens: sodium azide (TA1535), 4-nitroquinolene-*N*-oxide (TA97a and TA98), methyl methanesulfonate (TA100 and TA102), and with metabolic activation by 2-aminoanthracene (TA1535) and 2-aminofluorene (TA97a, TA98, TA100). For testing of extracts II–IV, positive controls were performed without metabolic activation by using: sodium azide (TA100 and TA1535), 4-nitro-ο-phenylene-diamine (TA98, TA1537), methyl methanesulfonate (TA102), and with metabolic activation by 2-aminoanthracene (TA98, TA100, TA1535, TA1537, and TA102). The negative control consisted of solvent alone (Solv Ctrl). The selection of positive controls for particular strains strictly followed Guideline 471 of the OECD [20].

For the evaluation of the results, an increase of revertants was considered to be biologically relevant if the number of revertants was at least twofold of the solvent control for TA98, TA100, and TA102 and threefold of the solvent control for TA1535 and TA1537; in test I, for all strains, a twofold increase was considered to be relevant.

### 5.6. Statistics

Parameters measured in acute and repeated-dose toxicity tests which were characterized by continuous data were exposed to Bartlett’s test to meet the homogeneity of variance before conducting analysis of variance (ANOVA) and Dunnett’s test. Data which failed to meet the homogeneity criteria were analysed with the Student´s *t*-test. A *p*-value < 0.05 was considered as statistically significant.

## Figures and Tables

**Table 1:** Phytochemical characteristics of the *Sideritis scardica* extracts no. I–IV.

Extract no.	I	II	III	IV
Extraction solvent	Ethanol 20% *v*/*v*	Water	Ethanol 50% *v*/*v*	Heptane
Total polyphenols (%)	6.25	5.07	6.23	0.18
Flavonoids (%)	1.18	0.59	2.03	n.d.
Acteoside (%)	0.41	0.12	0.94	n.d.
Caffeoylquinic acids (%)	0.47	0.24	0.38	n.d.

n.d.—not detected.

**Table 2:** The hematological parameters (mean ± SD) measured at the end of dosing of *S. scardica* extract I (20% EtOH) on day 29 or day 43 in the case of the reversal group.

Parameter	Sex M/F	Group					
		0 (Control)	250	500	1000	0 (Reversal)	1000 (Reversal)
Hb (g/dL)	M	15.0 ± 1.1	15.2 ± 0.4	16.5 ± 1.5	14.5 ± 1.6	16.3 ± 0.5	16.7 ± 2.2
	F	15.6 ± 1.0	16.1 ± 0.8	15.5 ± 1.4	16.3 ± 0.7	15.8 ± 0.6	15.3 ± 0.5
RBC (× 10^6^/µL)	M	7.36 ± 0.67	7.15 ± 0.52	8.02 ± 0.65	6.65 ± 1.06	8.42 ± 0.25	8.48 ± 1.19
	F	7.62 ± 0.72	7.88 ± 0.20	7.51 ± 1.05	7.84 ± 0.33	7.97 ± 0.25	8.03 ± 0.29
HCT (%)	M	44.5 ± 3.1	45.1 ± 1.06	48.8 ± 4.2	43.4 ± 4.3	69.0 ± 2.5	70.0 ± 9.4
	F	45.3 ± 3.0	47.0 ± 2.0	45.6 ± 4.3	46.5 ± 2.4	65.6 ± 3.0	64.0 ± 1.0
MCV (fL)	M	60.6 ± 2.7	63.4 ± 3.6	60.1 ± 2.4	66.5 ± 3.7	81.9 ± 2.0	82.6 ± 1.0
	F	59.5 ± 1.8	59.7 ± 3.3	61.1 ± 4.1	59.4 ± 2.1	82.2 ± 1.6	79.6 ± 2.1
MCH (pg)	M	20.5 ± 1.3	21.3 ± 1.2	20.5 ± 0.7	21.9 ± 1.0	19.4 ± 0.5	19.8 ± 0.7
	F	20.5 ± 0.7	20.4 ± 1.3	20.8 ± 1.5	20.8 ± 0.8	19.9 ± 0.3	19.1 ± 0.7
MCHC (g/dL)	M	33.8 ± 0.6	33.7 ± 0.4	33.7 ± 0.4	32.9 ± 0.8	23.7 ± 0.3	23.9 ± 0.5
	F	34.5 ± 0.2	34.1 ± 0.3	34.0 ± 0.2	35.0 ± 0.3	24.2 ± 0.2	24.0 ± 0.5
PLT (× 10^3^/µL)	M	311.2 ± 29.9	370.6 ± 57.2	367.2 ± 100.4	358.8 ± 45.7	291.2 ± 128.0	254.4 ± 62.6
	F	316.6 ± 89.3	314.0 ± 61.7	328.8 ± 31.1	426.8 ± 56.0	348.8 ± 20.6	306.8 ± 82.7
WBC (× 10^3^/µL)	M	16.18 ± 1.49	12.36 ± 3.96	11.66 ± 3.11 *	18.30 ± 1.57	15.30 ± 2.41	17.04 ± 5.47
	F	18.50 ± 4.97	17.32 ± 3.37	13.32 ± 3.00	11.82 ± 7.46	12.7 ± 1.96	15.28 ± 2.89
NEU (%)	M	19.4 ± 3.0	20.8 ± 4.2	19.8 ± 4.8	20.2 ± 4.1	21.0 ± 3.5	19.2 ± 4.2
	F	21.2 ± 4.5	19.2 ± 3.8	20.4 ± 3.9	20.8 ± 3.3	21.2 ± 3.6	21.6 ± 4.3
LYMPH (%)	M	77.0 ± 2.5	76.0 ± 2.7	77.0 ± 4.8	76.6 ± 3.6	76.2 ± 2.6	77.4 ± 3.8
	F	76.0 ± 4.7	77.6 ± 3.6	76.6 ± 4.1	75.8 ± 2.6	75.0 ± 3.4	75.4 ± 4.0
EOS (%)	M	1.0 ± 0.7	0.8 ± 0.8	0.8 ± 0.8	1.0 ± 0.7	0.6 ± 0.9	1.0 ± 1.0
	F	0.6 ± 0.5	0.8 ± 0.8	0.8 ± 0.8	1.2 ± 0.8	1.2 ± 0.8	1.0 ± 0.7
MONO (%)	M	2.6 ± 1.1	2.4 ± 1.1	2.4 ± 0.5	2.2 ± 0.8	2.2 ± 0.8	2.4 ± 0.5
	F	2.2 ± 0.8	2.4 ± 0.5	2.2 ± 0.8	2.2 ± 0.8	2.6 ± 0.5	2.0 ± 0.7
BASO (%)	M	0.0 ± 0.0	0.0 ± 0.0	0.0 ± 0.0	0.0 ± 0.0	0.0 ± 0.0	0.0 ± 0.0
	F	0.0 ± 0.0	0.0 ± 0.0	0.0 ± 0.0	0.0 ± 0.0	0.0 ± 0.0	0.0 ± 0.0
PT (sec)	M	15.4 ± 3.9	15.0 ± 3.6	15.6 ± 2.3	16.0 ± 2.5	14.8 ± 3.7	14.4 ± 3.0
	F	15.4 ± 3.8	15.0 ± 2.9	15.4 ± 4.0	15.2 ± 3.6	17.2 ± 4.2	14.2 ± 3.0

* *p* < 0.05; Hb—haemoglobin; RBC—red blood cells; HCT—haematocrit; MCV—mean corpuscular volume, MCH—mean corpuscular haemoglobin, MCHC—MCH concentration; PLT—platelets; WBC—white blood cells; PCT—plateletcrit; MPV—mean platelet volume; NEU—neutrophils; LYMPH—lymphocytes; MONO—monocytes, EOS—eosinophils; BASO—basophils; PT—prothrombin time.

**Table 3:** The biochemical parameters (mean ± SD) measured at the end of dosing of *S. scardica* extract I (20% EtOH) on day 29 or day 43 in the case of the reversal group.

Parameter	Sex M/F	Group					
		0 (Control)	250	500	1000	0 (Reversal)	1000 (Reversal)
Total protein (g/dL)	M	7.44 ± 0.39	7.60 ± 0.60	7.61 ± 0.50	7.50 ± 0.46	7.52 ± 0.26	7.49 ± 0.28
	F	7.47 ± 0.47	7.48 ± 0.45	7.58 ± 0.57	7.36 ± 0.36	7.72 ± 0.52	7.38 ± 0.35
Urea (mg/dL)	M	40.4 ± 8.2	40.0 ± 2.9	40.2 ± 6.6	35.8 ± 5.8	40.2 ± 5.5	39.8 ± 4.9
	F	40.8 ± 2.6	41.8 ± 6.8	42.4 ± 5.7	41.4 ± 5.2	39.2 ± 5.6	40.0 ± 6.9
ALT (IU/L)	M	36.2 ± 7.7	40.8 ± 7.7	38.0 ± 4.8	36.8 ± 9.6	37.6 ± 7.6	36.6 ± 4.3
	F	36.0 ± 6.6	37.0 ± 6.0	37.6 ± 7.9	38.6 ± 6.6	34.4 ± 8.8	34.4 ± 8.5
AST (IU/L)	M	60.2 ± 6.2	59.0 ± 7.3	61.6 ± 5.7	58.2 ± 7.0	58.6 ± 4.7	63.0 ± 4.2
	F	60.4 ± 6.7	65.6 ± 3.6	61.8 ± 7.7	57.0 ± 8.1	60.4 ± 7.5	63.4 ± 5.9
AP (IU/L)	M	74.2 ± 4.4	72.6 ± 7.5	73.8 ± 8.9	68.0 ± 8.7	69.2 ± 6.8	71.8 ± 6.2
	F	73.4 ± 4.3	77.6 ± 3.7	72.8 ± 8.7	68.6 ± 9.2	70.2 ± 7.6	70.2 ± 10.4
Glucose (mg/dL)	M	88.8 ± 11.8	87.6 ± 15.1	85.8 ± 6.5	89.0 ± 13.2	95.4 ± 80	92.8 ± 12.8
	F	85.4 ± 13.1	97.2 ± 5.9	95.2 ± 16.4	92.4 ± 10.7	87.2 ± 15.8	94.8 ± 10.5
Albumin (g/dL)	M	3.50 ± 0.33	3.69 ± 0.37	3.56 ± 0.27	3.63 ± 0.33	3.48 ± 0.44	3.44 ± 0.12
	F	3.57 ± 0.29	3.47 ± 0.36	3.49 ± 0.38	3.51 ± 0.17	3.50 ± 0.35	3.38 ± 0,17
Globulin (g/dL)	M	3.94 ± 0.50	3.91 ± 0.49	4.05 ± 0.35	3.87 ± 0.51	4.04 ± 0.41	4.05 ± 0.28
	F	3.90 ± 0.45	4.01 ± 0.70	4.09 ± 0.59	3.85 ± 0.44	4.30 ± 0.76	4.0 ± 0.44
Creatinine (mg/dL)	M	1.07 ± 0.13	0.97 ± 0.13	0.96 ± 0.14	0.96 ± 0.10	0.97 ± 0.16	0.97 ± 0.09
	F	1.05 ± 0.18	0.99 ± 0.16	0.90 ± 0.04	1.05 ± 0.11	0.97 ± 0.09	0.93 ± 0.10
Total bilirubin (mg/dL)	M	0.63 ± 0.04	0.62 ± 0.12	0.62 ± 0.04	0.64 ± 0.11	0.65 ± 0.08	0.61 ± 0.10
	F	0.61 ± 0.11	0.58 ± 0.08	0.59 ± 0.08	0.59 ± 0.08	0.61 ± 0.07	0.67 ± 0.16
Total cholesterol (mg/dL)	M	58.6 ± 7.7	59.4 ± 6.3	57.6 ± 6.7	55.8 ± 5.3	60.2 ± 6.3	60.8 ± 4.6
	F	60.4 ± 7.4	61.0 ± 6.5	57.4 ± 5.7	57.6 ± 5.3	63.2 ± 5.4	58.4 ± 5.7
Triglycerides (mg/dL)	M	110.0 ± 8.4	106.8 ± 10.5	109.0 ± 8.7	108.8 ± 8.8	108.6 ± 10.3	111.2 ± 11.2
	F	107.4 ± 11.6	103.6 ± 7.7	103.0 ± 8.5	102.0 ± 12.0	108.0 ± 13.0	114.0 ± 8.7
Phosphorus (mg/dL)	M	4.10 ± 0.49	4.16 ± 0.70	4.34 ± 0.43	3.84 ± 0.71	3.86 ± 0.59	3.92 ± 0.61
	F	4.10 ± 0.63	3.88 ± 0.73	4.08 ± 0.54	3.66 ± 0.49	4.02 ± 0.66	3.76 ± 0.42
Calcium (mmol/L)	M	2.34 ± 0.11	2.88 ± 0.13 **	3.00 ± 0.38 **	2.99 ± 0.13 **	2.99 ± 0.13	4.15 ± 0.14 *
	F	3.95 ± 0.1	3.72 ± 0.12 **	3.86 ± 0.09	3.63 ± 0.08 **	3.63 ± 0.2	3.67 ± 0.13
Sodium (mmol/L)	M	144.5 ± 1.6	151.5 ± 1.4	150.7 ± 2.8	154.4 ± 2.2	161.8 ± 1.0	162.1 ± 1.6
	F	150.5 ± 1.2	148.7 ± 1.7	147.9 ± 1.1 *	147.8 ± 1.4 *	151.8 ± 1.1	151.9 ± 1.5
Potassium (mmol/L)	M	4.74 ± 0.34	4.73 ± 0.4	4.94 ± 0.2	5.00 ± .25	3.98 ± 0.3	4.05 ± 0.3
	F	4.95 ± 0.7	4.67 ± 0.3	4.66 ± 0.2	4.52 ± 0.4	4.08 ± 0.4	3.90 ± 0.3
Chloride (mmol/L)	M	100.7 ± 2.9	91.5 ± 2.1	91.8 ± 2.9	95.6 ± 3.5	109.7 ± 1.9	110.9 ± 1.6
	F	89.6 ± 2.9	91.1 ± 2.4	89.7 ± 1.0	89.3 ± 1.4	98.4 ± 1.4	99.2 ± 1.3

* *p* < 0.05; ** *p* < 0.01; AST—aspartate transaminase; ALT—alanine transaminase; AP—alkaline phosphatases.
